# Prevalence of bovine paratuberculosis in Chinese cattle populations: a meta-analysis

**DOI:** 10.3389/fcimb.2024.1424170

**Published:** 2024-11-21

**Authors:** Zhang Huiying, Chu Mingfeng, Cheng Wei, Chen Shuiyun, Liang Yuchen, Wang Honghai, Chen Xuelong, Qi Yanping

**Affiliations:** ^1^ Anhui Province Key Laboratory of Animal Nutritional Regulation and Health, Anhui Science and Technology University, Fengyang, China; ^2^ Daqing Agricultural and Rural Bureau, Daqing, Heilongjiang, China

**Keywords:** cattle, meta-analysis, prevalence, bovine paratuberculosis, China

## Abstract

**Background:**

Bovine paratuberculosis is a chronic infectious disease of ruminants primarily caused by *Mycobacterium avium subsp. paratuberculosis* (MAP). It is essentially a chronic granulomatous enteritis characterized by intractable diarrhea, progressive lethargy, and thickening of the intestinal mucosa with the formation of crumpled pouches. Bovine paratuberculosis not only adversely affects milk production and the quality of dairy products but also poses a significant threat to the economic development of dairy farming and human food security. This systematic review and meta-analysis was conducted to assess the prevalence of MAP infection among cattle herds in mainland China

**Results:**

A total of 62 studies with data from 102,340 cattle in 24 provinces in China were selected after matching the assessment criteria. In China, the overall estimated prevalence of MAP infection in cattle was 8%(7727/102340). Interestingly, the MAP infection rate in cattle in southern China was estimated to be 2% (6/281), which was significantly lower compared with other regions of China, and the highest infection rate was 12% (1914/16008) in eastern China. MAP infection rates were related to age, average herd size, type of use, season, detection method, and sample type. Moreover, the MAP infection rate in cattle did not correlate with the publication date of the studies.

**Conclusion:**

The analysis identified age, average herd size, type of use, and season as significant potential risk factors associated with PTB pool positivity. In addition, the detection method and sample type can also potentially affect the incidence of detected PTB.

## 1 Introduction

Paratuberculosis (PTB) is a chronic infectious disease that mainly affects ruminants and is caused by *Mycobacterium avium subsp. paratuberculosis* (MAP) (Fecteau, 2018). First reported in 1894, the disease, also known as Johne’s disease (JD), was discovered by Johne et al. and subsequently classified as a Notifiable Animal Disease by the Office International des Epizooties (OIE) (Hussein, 2021). The main clinical symptoms associated with MAP infection in cattle include persistent diarrhea. In the early stages of infection, animals maintain a normal body temperature and show no significant change in food intake (Li, 2019). However, intermittent diarrhea is observed as the disease worsens, accompanied by emaciation, and eventually transforms into refractory diarrhea. Chronic progressive weight loss and chronic or intermittent diarrhea serve as the primary clinical indicators of PTB in cattle (Chiodini et al., 1984). MAP infection in cattle can lead to fatality rates as high as 10% (Wang et al., 2021a). The disease can spread widely and the feces of infected cattle contain a large number of pathogenic bacteria, which can contaminate pastures and feed (Liyuan, 2018). Vaccination against paratuberculosis can lead to positive results in tests for the disease, presenting a challenge for large-scale immunization. Reports indicate that MAP-positive herds incur nearly $100 more in losses per cow than MAP-negative herds (Cheng et al., 2020). The main economic losses caused by bovine paratuberculosis are premature slaughter, substantially reduced milk production, and weight loss in infected cattle (Hasonova and Pavlik, 2006). The economic impact of paratuberculosis in the USA is considerable, with annual losses exceeding $200 million (Groenendaal et al., 2015). Therefore, bovine paratuberculosis has attracted significant attention in recent years. This study provides critical reference data for understanding and analyzing the epidemiological characteristics of bovine PTB in Chinese cattle.

In China, the first case of paratuberculosis was reported in Inner Mongolia in 1953, followed by outbreaks in Heilongjiang, Jilin, and Hebei Provinces, covering nearly all of northern China and resulting in the classification of the PTB as a secondary animal disease. In 1974, two cases of PTB were reported in the reclamation area of Xinjiang Agricultural Division No. 3 Shui Gong Regiment (Libo et al., 2004). In 1981, it was reported that approximately 4.4% of the cattle in Heilongjiang Province were infected (Kwan et al., 1981). In addition, by 2000, 1250 cattle were tested using enzyme-linked immunosorbent assay (ELISA) in Shanghai, with nine found to be positive (Yongkang et al., 2000). However, with the continuous development of intensive large-scale cattle farms, the rate of PTB positivity has increased annually, reaching up to 56% in Ningxia in 2019 (Cheng et al., 2019), thereby causing huge losses to the farming industry in China. It is necessary to analyze and summarize the overall situation of MAP infection in China.

Given the relatively fragmented nature of epidemiological investigations of bovine PTB, this study adopted a meta-analytic approach to analyze the relevant literature on the epidemiological investigations of bovine PTB in China between 1981 and 2022. Moreover, the statistics related to age, average herd size, type of use, detection method, sample type and season were used to further understand the epidemiological trends and targeted prevention and control of bovine PTB and to provide ideas for future research on the disease. This study aimed to estimate the prevalence of MAP antibodies in cattle herds in China and provide ideas for analyzing the prevalence of PTB in cattle worldwide.

## 2 Methods

### 2.1 Search strategy

Based on the MOOSE guideline (Stroup et al., 2000), a comprehensive search was conducted for studies published between 1981 and 2022 on the epidemiology of bovine PTB in China, using various Chinese and English-language databases, including PubMed, CNKI, VIP, Cochrane Library, ScienceDirect, Web of Science, Google Scholar, Clinical Trials, and Wanfang, to identify relevant articles. The search terms included “MAP or PTB or JD or paratuberculosis or bovine paratuberculosis or bovine diarrheal disease,” “epidemiology or incidence or prevalence,” “Cattle or Bovine or Ruminant,” and combinations of these phrases.

### 2.2 Data management

Two investigators independently evaluated the extracted data to determine whether they met the inclusion criteria, and studies that met the requirements were ultimately included. In cases of discrepancies during the screening process, expert consultation and analysis were sought to determine study inclusion. This research involved a statistically controlled primary survey where data were extracted directly from the articles without further verification from the authors. The study also did not include unpublished data.

### 2.3 Exclusion criteria

Publications with the following criteria were excluded:

- Studies related to animals other than the cattle;
- Sampling conducted outside mainland China or sample size with fewer than 30 cows;
- Secondary research;
- Undisclosed data sample size.

### 2.4 Inclusion criteria

- Publications with the following criteria were included:
- Studies related to cattle studies;
- The sampling location was in mainland China;
- The sample size should be more than 30 cattle;
- Studies must have reported any epidemiological data related to MAP or bovine PTB;
- Studies with time of data collection, sample size, or denominator for each reported prevalence or rate.

### 2.5 Data extraction

A standardized data collection form was used for data extraction. The information recorded was as follows: first author, year of publication, province of investigation, method of testing (e.g., ELISA, PCR, intradermal paratuberculin tests), total number of cattle tested and number of MAP-positive cattle, feeding stage or age, season of investigation, breed of cattle, sample type and the average herd size.

### 2.6 Analysis of study quality

**Figure 1:**
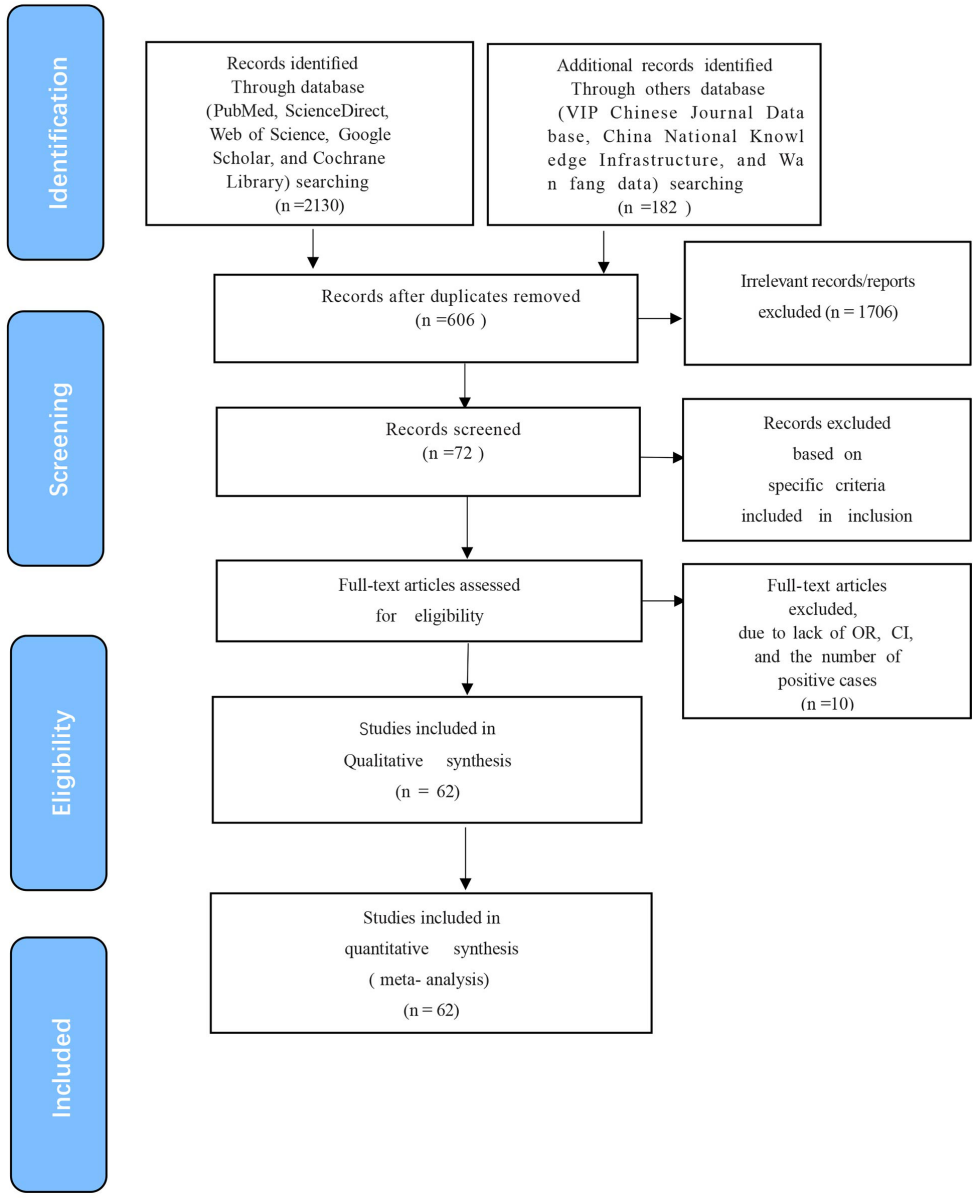
Flow diagram showing the selection of eligible studies. A total of 62 articles were obtained by screening the database in four processes: “Identification”, “Screening”, “Eligibility” and “Included”.

The quality of the studies was evaluated according to the Grading of Recommendations Assessment, Development, and Evaluation (GRADE) (Guyatt et al., 2008). The scoring for each subgroup was determined based on criteria that included stating the study’s purpose, detection method, sampling time, sample collection details, and risk factor analysis. Studies scoring 4-5 were considered high quality, those scoring 2-3 were deemed moderate quality, and those scoring 1 or less were categorized as low quality (**Figure 1**).

### 2.7 Statistical analysis

Meta-analysis was conducted to calculate the overall prevalence of bovine PTB infection and assess heterogeneity using Stata 12 software (Stata Corp.College Station, Texas); results, including 95% confidence intervals, were presented in forest plots. Heterogeneity was evaluated using I^2^ and Cochrane Q (represented as χ² and P values) statistics, as detailed in **Table 1**. Subgroup and regression analyses were conducted to investigate potential sources of heterogeneity when statistical disparities were observed In this study, different factors affecting heterogeneity were analyzed separately and established in a multivariate model, which examined the role of factors such as province of investigation, assay method, season of investigation, feeding stage or age, sample type, breed of cattle, and average herd size and potential publication bias was assessed by funnel plots (Gong et al., 2021). The meta-analysis was conducted according to the Preferred Reporting Items for Systematic Evaluation and Meta-Analyses (PRISMA) guidelines (Moher et al., 2015), ensuring the quality of this review’s presentation. Apparent prevalence (AP) was calculated as AP = number of positive tests/total number of tests. True prevalence (TP) was adjusted for test sensitivity (Se) and specificity (Sp) and is detailed in **Table 2**. TP was computed using the formula TP = (AP+Sp-1)/(Se+Sp-1), with the sensitivity of the ELISA test set at 98.0% and specificity at 99.5%. The Se was 98.0%, and the Sp was 99% for the test of positivity using the PCR method in relevant studies (Bao et al., 2017; Zhang et al., 2024). The Se and Sq of the intradermal test for paratuberculin were 80% and 96%, respectively (Han et al., 2014).

**Table 1:** Pooled prevalence of MAP infection in cattle in mainland China.

		No. studies	No. tested	No. positive	% (95% CI)	Heterogeneity		
						χ²	P-value	I² (%)
Region	Northeast China	11	30202	1872	13% (8-19)	1179.30	0.000	99.2%
	North China	19	30972	2169	7% (5-9)	1357.72	0.000	98.7%
	Northwest China	16	9302	857	6% (4-8)	497.62	0.000	96.6%
	East China	13	16008	1914	7% (5-9)	903.68	0.000	98.7%
	South China	1	281	6	8% (6-9)	0	0.000	0.0%
	Central China	9	10051	497	6% (3-9)	953.69	0.000	99.1%
	Southwest China	7	5134	413	15% (3-27)	591.73	0.000	99.2%
Age	calf	13	2985	214	7% (9-18)	357.88	0.000	96.9%
	Youth cattle	5	18226	785	2% (1-6)	256.53	0.000	98.4%
	Adult cattle	15	17815	2752	15% (9-18)	1803.34	0.000	99.2%%
	Not Found	46	63314	3977	6% (7-9)	3351.40	0.000	98.7%
Detection method	ELISA	53	91239	6211	7% (8-10)	4170.18	0.000	98.8%
	Intradermal tests of paratuberculin	9	6915	593	9% (5-11)	723.01	0.000	99.0%
	PCR	9	1357	233	17% (14-34)	212.43	0.000	96.2%
	Other	6	2829	691	24% (19-28)	22.01	0.000	81.8%
Average herd size	<500cattle	12	7138	578	8% (7-13)	406.68	0.000	97.3%
	500-1000 cattle	6	10005	1471	15% (13-30)	694.06	0.000	99.3%
	>1000 cattle	11	21680	1238	6% (10-18)	728.8	0.000	98.8%
	Not Found	51	63517	4441	7% (7-10)	3867.46	0.000	98.8%
Sample type	Serum	61	100968	7477	7% (8-10)	6010.25	0.000	99.0%
	Other	11	1372	251	18% (14-33)	220.03	0.000	95.9%
	Beef cattle	13	4617	272	6% (4-9)	206.81	0.000	95.2%
Type of use Season	Dual-purpose cattle	7	3347	117	3% (1-5)	65.87	0.000	90.9%
	Dairy cow	44	84102	6766	8% (10-12)	5464.36	0.000	99.2%
	Not Found	13	10275	573	6% (5-9)	561.1	0.000	97.9%
	Spring	3	862	54	6% (2-11)	12.48	0.02	84.0%
	Summer	4	2866	124	4% (2-6)	11.53	0.09	74.0%
	Autumn	7	11102	1027	9% (6-12)	146.76	0.000	95.9%
	Winter	3	2121	190	9% (1-22)	245.67	0.000	99.2%
	Not Found	50	85389	6333	7% (9-11)	5272.21	0.000	99.1%

ELISA, enzyme-linked immunosorbent assay; PCR, Polymerase Chain Reaction; CI, Confidence interval.

P-value < 0.05 is statistically significant.

Region: Central China: Henan; Eastern China: Anhui, Shandong, Shanghai, Jiangxi, Jiangsu, Zhejiang; Northern China: Beijing, Hebei, Inner Mongolia, Shanxi;

Northeastern China: Heilongjiang, Liaoning, Jilin; Northwestern China: Gansu, Ningxia, Qinghai, Shaanxi, Xinjiang; Southern China: Fujian, Guangxi; Southwestern.

China: Guizhou, Sichuan, Tibet, Yunnan.

Age: calves (0-12 MoA/months of age), young cattle (13-24MoA/months of age), adult cattle (>24MoA/months of age).

**Table 2:** Included studies of MAP infection in cattle in mainland China.

Study ID	Province	Region	No. examined	No. positive	Apparent Prevalence	Detection method	True Prevalence	95%CI (upper and lower limits)	Study design	Quality score
(Hu et al., 2021)	Shanghai	East China	1260	26	2%	ELISA	1.6%	(0.01, 0.03)	Cross-sectional	4
(Tang et al., 2020)	Hunan	Central China	450	21	5%	ELISA	4.3%	(0.03, 0.07)	Cross sectional	4
(Chen et al., 2019)	Beijing.	North China	8549	791	9%	ELISA	9.0%	(0.09, 0.10)	Cross-sectional	7
(Liu et al., 2016)	Hebei	North China	4279	25	1%	ELISA	0.1%	(0.00, 0.01)	Cross sectional	4
(Gao and Kang, 2017)	Ningxia	Northwest China	1796	77	4%	ELISA	3.9%	(0.03, 0.05)	Cross sectional	5
(Cheng et al., 2016)	Shandong	East China	420	9	2%	PCR	1.5%	(0.01, 0.04)	Cross sectional	5
(Mehmeti, 2021)	Xinjiang	Northwest China	1710	81	5%	ELISA	4.3%	(0.04, 0.06)	Cross sectional	6
(Li, 2014)	Inner Mongolia	North China	2391	336	14%	ELISA	13.9%	(0.13, 0.15)	Cross sectional	5
(Lin, 2015)	Shandong	East China	736	35	5%	ELISA	4.4%	(0.03, 0.06)	Cross sectional	4
(Yue et al., 2016)	Shandong	East China	1038	121	12%	ELISA	11.4%	(0.10, 0.14)	Cross sectional	4
(P. Quan et al., 2022)	Henan etc	Central China	432	18	4%	ELISA	3.8%	(0.02, 0.06)	Cross sectional	5
(Sun et al., 2015)	Hebei etc	North China	3600	82	2%	ELISA	1.8%	(0.02, 0.03)	Cross sectional	4
(Zhang Z., 2020)	Xinjiang	Northwest China	695	94	14%	ELISA	13.4%	(0.11, 0.16)	Cross sectional	3
(Abdulmigeti et al., 2019)	Xinjiang	Northwest China	80	4	5%	ELISA	4.6%	(0.00, 0.10)	Cross sectional	5
(Zhang et al., 2020)	Xinjiang	Northwest China	114	5	4%	ELISA	4.0%	(0.01, 0.08)	Cross sectional	4
(Mehmetimin, 2022)	Xinjiang	Northwest China	1003	76	8%	ELISA	7.3%	(0.06, 0.09)	Cross sectional	6
(Sun and Liu, 2019)	Xinjiang	Northwest China	114	5	4%	ELISA	4.0%	(0.01, 0.08)	Cross sectional	4
(Wang et al., 2015)	Tibet	Southwest China	468	9	2%	ELISA	1.5%	(0.01, 0.03)	Cross sectional	4
(Pi et al., 2014)	Guizhou	Southwest China	920	275	30%	ELISA	28.3%	(0.27, 0.33)	Cross sectional	5
(Tian et al., 2014)	Inner Mongolia	North China	2391	336	14%	ELISA	13.9%	(0.13, 0.15)	Cross sectional	5
(Tan et al., 2015)	Guangxi	South China	281	6	2%	ELISA	1.7%	(0.00, 0.04)	Cross sectional	5
(Han et al., 2014)	Shandong	East China	3356	870	26%	ELISA	24.1%	(0.24, 0.27)	Cross sectional	5
(Chen et al., 2022)	Shandong	East China	3253	579	18%	ELISA	17.7%	(0.16, 0.19)	Cross sectional	6
(He et al., 2019)	Liaoning	Northeast China	860	48	6%	ELISA	5.2%	(0.04, 0.07)	Cross sectional	5
(Yan et al., 2018)	Shanghai	East China	150	20	13%	ELISA	13.2	(0.08, 0.19)	Cross sectional	5
(Cheng et al., 2019)	Ningxia	Northwest China	189	106	56%	ELISA	55.8%	(0.49, 0.63)	Cross sectional	5
(Shi et al., 2020)	Heilongjiang	Northeast China	15035	680	5%	ELISA	4.1%	(0.04, 0.05)	Cross sectional (Chi, 2007)	5
(Liu et al., 2015)	Sichuan etc	Southwest China	921	17	2%	ELISA	1.4%	(0.01, 0.03)	Cross sectional	4
(Zhang K., 2020)	Hunan	Central China	450	21	5%	ELISA	4.3%	(0.03, 0.07)	Cross sectional	4
(Qu et al., 2015)	Shanghai	East China	430	16	4%	ELISA	3.3	(0.02, 0.06)	Cross sectional	5
(Zhang et al., 2022)	Xinjiang	Northwest China	263	48	18%	ELISA	16.1%	(0.14, 0.23)	Cross sectional	5
(Xing, 2018)	Henan	Central China	480	27	6%	ELISA	5.3%	(0.04, 0.08)	Cross sectional	6
(Chang et al., 2020)	Gansu	Northwest China	227	13	6%	ELISA	5.3%	(0.03, 0.09)	Cross sectional	4
(He et al., 2016)	Gansu	Northwest China	280	1	1%	ELISA	0.7%	(-0.00, 0.01)	Cross sectional	5
(Zhao et al., 2020)	Beijing	North China	1149	153	13%	ELISA	11.8%	(0.11, 0.15)	Cross sectional	4
(Wang et al., 2000)	Shanghai	East China	1250	9	1%	ELISA	0.2%	(0.00, 0.01)	Cross sectional	5
(Xue and Li, 2020)	Heilongjiang	Northeast China	6139	351	6%	Intradermal tests of paratuberculin	2.3%	(0.05, 0.06)	Cross sectional	4
(Cai et al., 1981)	Heilongjiang	Northeast China	91	4	4%	ELISA	4%	(0.00, 0.09)	Cross sectional	5
(Chi, 2007)	Jilin	Northeast China	300	93	31%	ELISA	31.3%	(0.01, 0.06)	Cross sectional	4
(Zhang, 2018)	Hebei	North China	233	9	4%	ELISA	3.4%	(0.23, 0.29)	Cross sectional	4
(Han, 2018)	Shanxi	North China	784	202	26%	ELISA	25.9%		Cross sectional	7
(Wu et al., 2020)	Xinjiang	Northwest China	965	297	31%	ELISA	31.1%	(0.28, 0.34)	Cross sectional	5
(Chao et al., 2021)	Liaoning	Northeast China	3070	193	6%	ELISA	5.9%	(0.05, 0.07)	Cross sectional	4
(Wang et al., 2021b)	Xinjiang	Northwest China	201	14	7%	PCR	5.1%	(0.03, 0.10)	Cross sectional	4
(Jin et al., 2022)	Heilongjiang	Northeast China	900	30	3%	ELISA	2.9%	(0.02, 0.05)	Cross sectional	3
(Lin, 2012)	Beijing	North China	326	72	22%	PPD	23.8%	(0.18, 0.27)	Cross sectional	7
(Zhang, 2008)	Yunnan	Southwest China	569	2	0.4%	PCR	0%	(-0.00, 0.01)	Cross sectional	3
(Zhang, 2016)	Shandong	East China	900	97	11%	ELISA	3.8%	(0.09, 0.13)	Cross sectional	3
(Li, 2019)	Henan	Central China	305	13	4%	other	None	(0.02, 0.07)	Cross sectional	5
(Jingle et al., 1981)	Jilin	Northeast China	1244	285	23%	ELISA	23.0%	(0.21, 0.25)	Cross sectional	4
(Cheng et al., 2016)	Shandong	East China	586	83	14%	ELISA	14.0%	(0.11, 0.17)	Cross sectional	5
(Liang, 1985)	Hebei	North China	39	9	23%	ELISA	23.2	(0.10, 0.36)	Cross sectional	4
(Wang XP. et al., 2021)	Henan	Central China	868	249	29%	Intradermal tests of paratuberculin	32.5%	(0.26, 0.32)	Cross sectional	5
(Yan et al., 2020)	Henan	Central China	305	13	4%	ELISA	3.9%	(0.02, 0.07)	Cross sectional	5
(Liu, 1996)	Inner Mongolia	North China	354	8	2%	PCR	0.3%	(0.01, 0.04)	Cross sectional	7
(Zhong et al., 2005)	Heilongjiang	North China	358	10	3%	Intradermal tests of paratuberculin	0%	(0.01, 0.05)	Cross sectional	5
(Liu et al., 1995)	Heilongjiang	North China	924	128	14%	ELISA	13.3%	(0.12, 0.16)	Cross sectional	3
(Zhu et al., 1999)	Beijing	North China	232	7	3%	Intradermal tests of paratuberculin	0%	(0.01, 0.05)	Cross sectional	5
(Wang et al., 2022)	Hebei	North China	114	18	16%	Intradermal tests of paratuberculin	15.5%	(0.09, 0.22)	Cross sectional	3
(Gao et al., 1997)	Inner Mongolia	North China	1720	90	5%	PCR	3.4%	(0.04, 0.06)	Cross sectional	5
(Zhu et al., 1997)	Beijing	North China	3235	14	0.4%	Intradermal tests of paratuberculin	0%	(0.00, 0.01) (0.08, 0.10)	Cross sectional	4

ELISA, enzyme-linked immunosorbent assay; PCR, polymerase chain reaction.

## 3 Results

### 3.1 Selected studies on PTB in cattle

**Figure 2:**
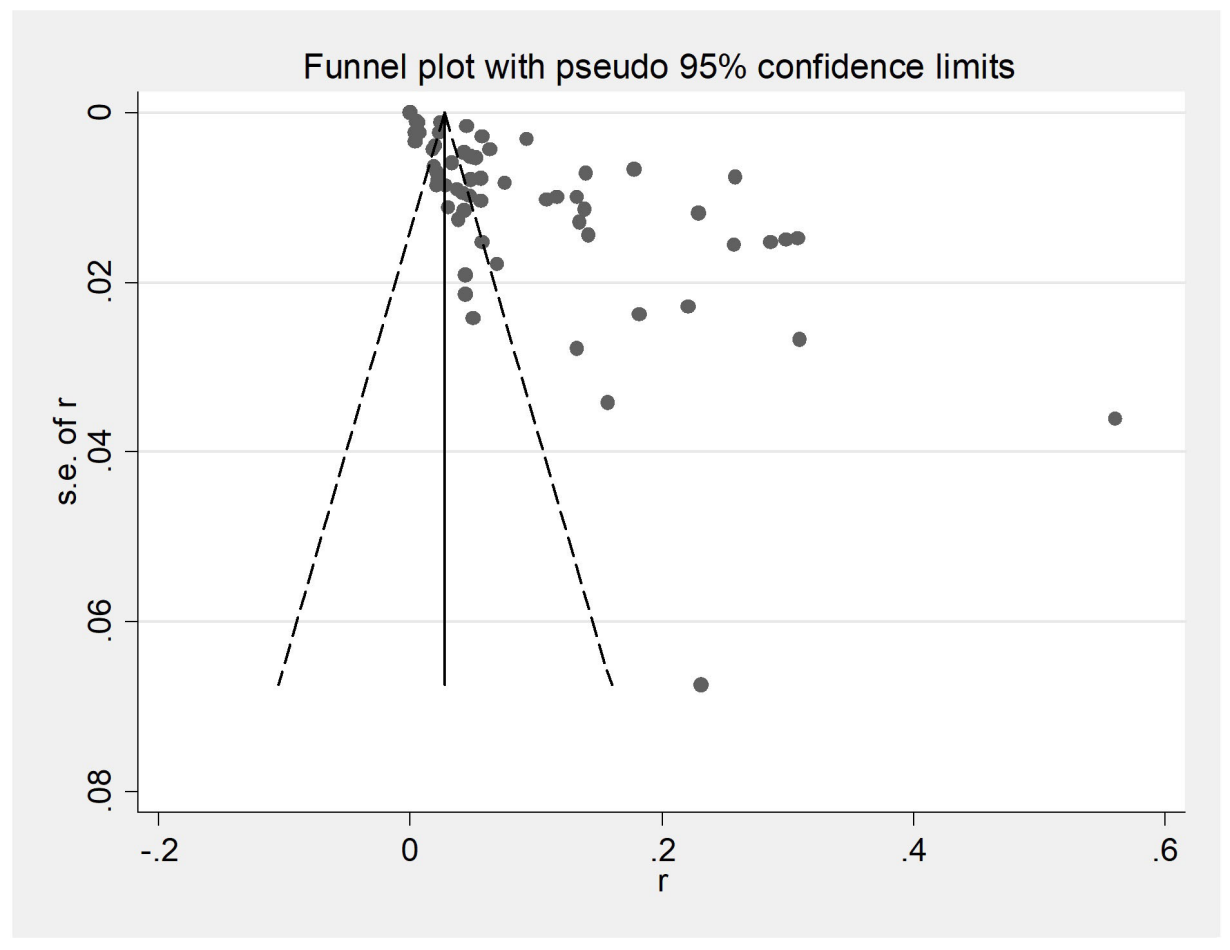
Funnel plot with pseudo 95% confidence interval limits for the examination of publication bias. A funnel plot is a scatter plot based on the effect size of each experimental study as the horizontal coordinate and the sample size as the vertical coordinate for each experimental study. At the top of the funnel plot and enriched towards the center are samples with high learning volume and high accuracy; at the bottom and scattered towards the periphery are samples with small learning volumes and low accuracy.

The initial database search identified 2312 articles, which was narrowed down to 72 after removing duplicates and performing initial screening. Ten articles were further excluded due to small sample sizes (fewer than 30 cattle in 2 articles), inconsistent or incomplete data (4 articles), insufficient detail (1 article provided only prevalence), and irrelevance to cattle (3 articles). Thus, finally, 62 full-text studies were included for quantitative analysis (**Table 2**). The data were obtained from 102,340 head of cattle from 24 different provinces in China, including 30,202 in northeastern China, 9,302 in northwestern China, 5,134 in southwestern China, 30,972 in northern China, 16,008 in eastern China, 281 in southern China, and 10,051 in central China. Analysis revealed that Northern China had the highest survey count among the regions. Of the selected articles, 61 were in Chinese, and one was in English (**Table 1**). A total of 62 papers, with the earliest published in 1981, were included. A cross-sectional analysis of these papers was conducted to calculate the prevalence rates for each period. Based on the established quality criteria, 8 papers were categorized as high quality (4 or 5 points), 47 as medium quality (2 or 3 points), and 6 as low quality (1 point). Publication bias was assessed using a funnel plot (**Figure 2**), which displayed an asymmetric shape, suggesting potential publication bias.

### 3.2 The prevalence of PTB in various regions of China

**Figure 3:**
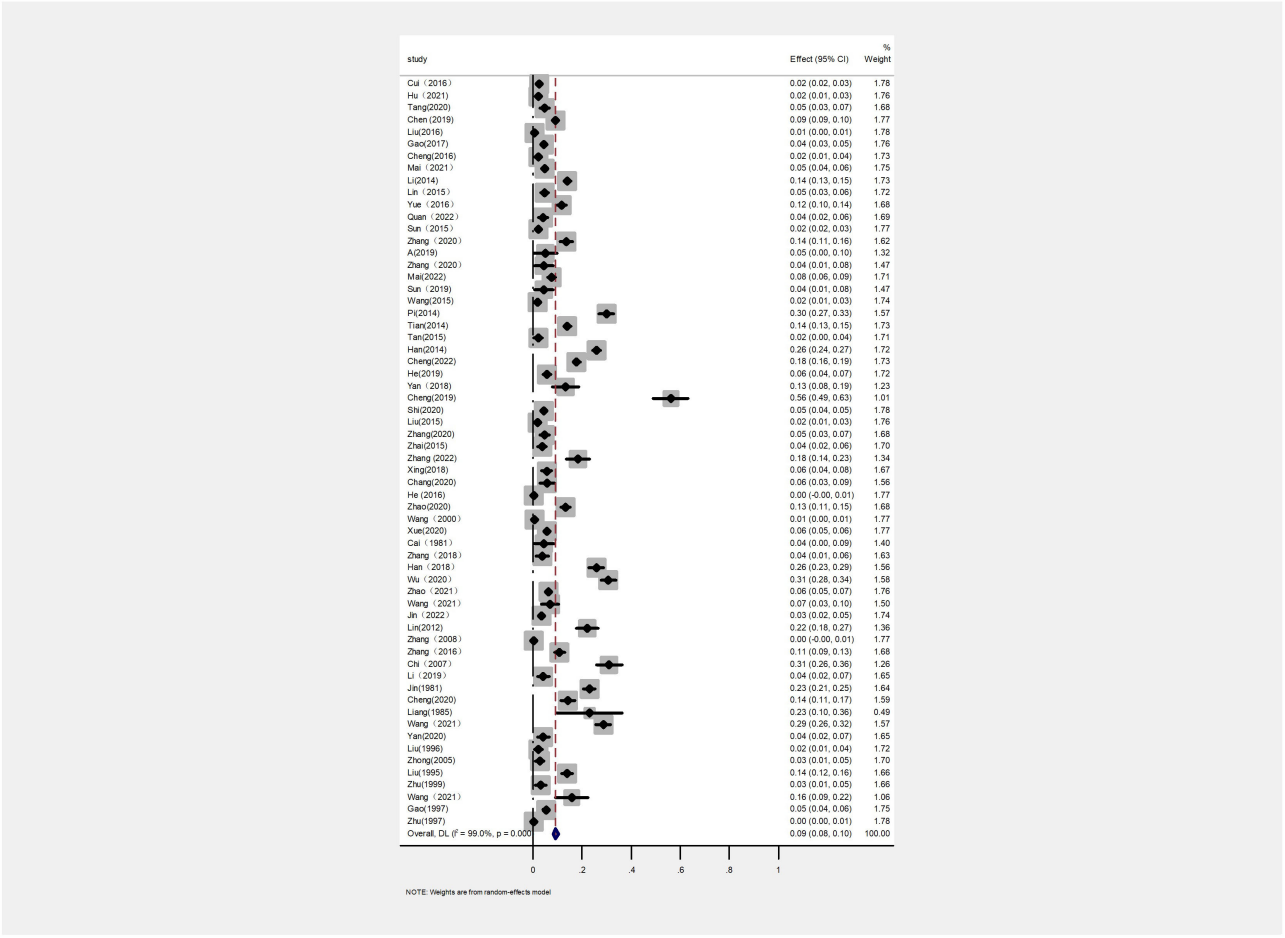
Forest plot of paratuberculosis pooled prevalence in cattle in China. The length of the horizontal line indicates the 95% confidence interval and the diamond indicates the summed effect.

**Figure 4:**
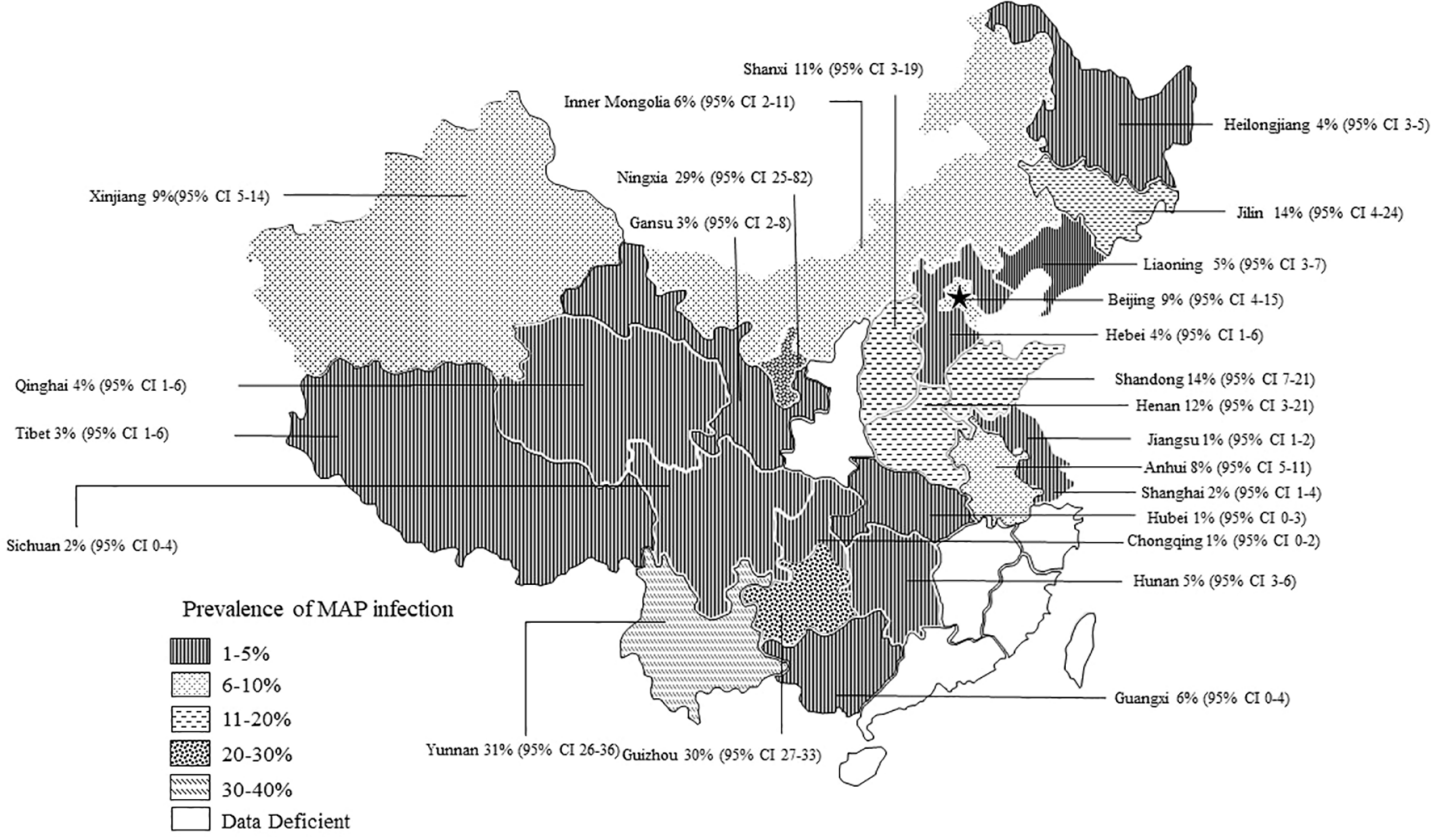
Map of bovine infection with pooled paratuberculosis in China. The pattern of squares in the lower-left corner of the graph is sequentially decreasing in prevalence and data deficient. Data Deficient: Indicates that there were no epidemiological surveys of PTB in the province during the statistical year. The labelled data are: Province [(pooled prevalence) 95% Conf. Interval].

The AP of bovine PTB across the studies ranged from 0.4% to 56% (**Figure 3**; **Table 2**), while the TP varied from 0% to 55.8%, showing high heterogeneity (I²=99%, P=0.000). For instance, Eastern China had the highest pooled prevalence of MAP at 12% (95% CI 5-7, 1914/16008), followed by Northwestern China at 9% (95% CI 4), and Southwestern, Northern, Northeastern, and Central China at 8% (95% CI 3-8, 413/5134), respectively. The pooled prevalence of MAP was 8% (95% CI 3-8, 413/5134), 7% (95% CI 5-9, 2169/30972), 6% (95% CI 8-19, 1872/30202), and 5% (95% CI 3-9, 497/10051) in Southwestern, Northwestern, Northeastern, and Central China, respectively (**Table 1**). In addition, the provincial subgroups showed that the positivity rates in Chinese provinces ranged from 1 to 31%, with Guizhou and Jilin displaying much higher pooled prevalence rates in comparison to other provinces at 30% (95% CI 27-33, 275/920) and 21% (95% CI 4-24, 506/2468), respectively. Jiangsu, Hebei, Chongqing, and Yunnan had the lowest pooled prevalence rates which were around 1% [Jiangsu 1% (95% CI 1-2,29/2379), Hebei 1% (95% CI 0-3,77/5465), Chongqing 1% (95% CI 0-2,3/400), and Yunnan 1% (95% CI 26-36,2/569)] (**Figure 4**).

### 3.3 Results of the meta-analysis

Age subgroup analysis indicated the highest pooled prevalence of 15% (95%CI 8-18, 2752/17815) in adult cattle (>24 months of age), compared to 2% (95%CI 1-6, 785/18226) in young cattle (13-24 months of age) and 7% (95%CI 9-18, 214/2985) in calves. The analysis showed that ELISA was the most commonly used MAP assay (7%, 95%CI 8-10, 6211/91239), followed by PCR (17%, 95%CI 14-34, 233/1357) and intradermal paratuberculin tests (9%, 95%CI 5-11, 593/6915). Additionally, in the subgroup analysis of average herd size, classified into <500 cows, 500-1000 cows, and >1000 cows, significantly {Zilong, 2020 #37}higher prevalence was observed in the 500-1000 cow group (15%, 95%CI 13-30, 1471/10005) than in the <500 cow group (8%, 95%CI 7-13, 578/7138) and the > 1000 cow group (6%, 95%CI 10-18, 1238/21680). The use of serum testing (7%, 95%CI 8-10, 7477/100968) was substantially more than other forms of sampling (18%, 95%CI 14-33, 251/1372) in the articles published in this survey. Moreover, analysis of the type of use showed that MAP infection was markedly more frequent in dairy cows (8%, 95%CI 10-12, 6766/84102) than in other varieties, with MAP infection in dual-purpose cattle (milk and beef production) and beef cattle being 3% (95%CI 1-5, 117/3347) and 6% (95%CI 4-9, 272/4617), respectively. Seasonal analysis indicated that MAP infection rates were highest in autumn and winter at 9% (95%CI 6-12, 1027/11102 for autumn; 95%CI 1-22, 190/2121 for winter), compared to lower rates in spring and summer at 6% (95%CI 2-11, 54/862) and 4% (95%CI 2-6, 124/2866), respectively (**Table 1**).

Although some bias was observed in the collected data, it was reliable and may provide a sound basis for investigating the prevalence of PTB in cattle in China.

## 4 Discussion

This systematic review and meta-analysis screened published literature on bovine PTB in China to evaluate factors influencing disease prevalence and to inform suitable control measures. Bovine PTB is a chronic granulomatous enteritis caused by *Mycobacterium avium subsp. paratuberculosis* and is widely prevalent in countries worldwide. The incidence of PTB has rapidly increased in China, with large-scale cattle farms reporting successive outbreaks. In addition, no specific drugs are available to manage the disease. Thus, in June 2022, the Ministry of Agriculture of the People’s Republic of China classified PTB as a secondary animal disease, which indicated that bovine PTB constitutes an important disease that requires long-term monitoring (Ministry of agriculture and rural affairs of the people’s republic of China announcement no. 573, 2022).

This review analyzed 62 studies published between 1981 and 2021, documenting an overall PTB prevalence of 8.0% among 102,340 cattle in China, slightly lower than reports from other countries. PTB is particularly common in countries with developed cattle industries, such as the United Kingdom, France, Denmark, the Netherlands, Germany, the USA, Russia, Australia, Canada, and New Zealand (Boelaert et al., 2000; Ssekitoleko et al., 2022). For instance, ELISA results in Colombia on asymptomatic cattle showed a 10% positivity rate for anti-MAP antibodies in asymptomatic cattle (Elmagzoub et al., 2020). The overall apparent prevalence of MAP infection was found to be 6.3% and 18.9% at animal and herd levels, respectively, in cattle in Khartoum State (Elmagzoub et al., 2020). In another study, Woodbine et al (Woodbine et al., 2009). reported an average individual positivity rate of 7.1% and an average farm positivity rate of 76% in 114 cattle farms using ELISA in southwestern England. However, there are fewer reports of PTB in Asia and the rates vary widely from country to country. Japan has a low prevalence, with about 1,000 of the 500,000 cattle officially examined in Japan diagnosed annually with PTB (Hasonova and Pavlik, 2006). The prevalence of PTB in this analysis was 8%, relatively lower than that observed in some European and American countries. For instance, Mongolia has a large cattle industry, and its PTB prevalence is only 0.84% (Hu et al., 2021).

Due to the complexity of the immune response against pathogens, different diagnostic methods have to be used based on the different infection periods because the suitability and sensitivity of the tests depend on the clinical stage of the disease (Whittington et al., 2017). In addition, the methods used to detect MAP infection have relative advantages and applications. In China, most studies have used ELISA serological methods, although a few have also used PCR and other microbiological techniques, which is consistent with a Brazilian study (Espeschit et al., 2017). For example, in a study reported by Ferreira et al., an ELISA (PPA) assay was used to test 179 cattle suspected to be positive for PTB. Interestingly, a study by Echeverr et al. reported that while the detection of MAP infection by ELISA was both easy and practical, it should be used only as a screening method to identify various animals sensitized by mycobacteria, while specific diagnosis using “reference standard” methods, such as the isolation of MAP or PCR detection of MAP DNA, should be used for the confirmation (Mbindyo et al., 2020). Most of the samples evaluated in the present meta-analysis were sera. Another method used to detect MAP involves the analysis of the feces; however, the MAP levels in feces tend to be very low (Osterstock et al., 2008), which may be why serum rather than feces was chosen for MAP testing in most studies.

Sample type significantly influences MAP assay variability, with varying sensitivities observed across different samples, including serum, milk, stool, and cadaver. Serum and milk are predominantly used for (indirect) serological tests (ELISA) detecting antibodies (Gupta et al., 2012), while feces and cadaver (Liu et al., 1995), in particular gut and associated lymph nodes (Bakker et al., 2000; Cheng et al., 2020), are tested with molecular biological methods. In this analysis, most of the samples tested for MAP in the included studies were sera, and a meta-analysis showed that the sensitivity of ELISA was significantly higher in serum samples than in necropsy samples (Collins MT et al., 2006). Overall, detecting antibodies using ELISA is considered the method of choice for diagnosing PTB for its speed and cost-effectiveness.

Seasonality significantly influences the PTB positivity rate. The analysis indicates higher PTB prevalence in autumn and winter compared to summer and spring. However, according to a report by Wolf et al (Wolf et al., 2015), samples collected in the spring and summer had a higher chance of testing positive for MAP than samples collected in the winter. This suggests that seasonal variations in temperature and humidity may affect the viability of MAP bacteria in environmental samples. This could be attributed to the influence of differences in climate between countries. According to Zare et al (Zare et al., 2013), both the season and the animals’ age also profoundly affect MAP infection. This is consistent with prior studies reported in China on the potential impact of seasonal factors on MAP infection rates.

The study revealed a higher pooled prevalence of PTB in calves (0-12 months of age) and adult cattle (>24 months of age) at 15% (95% CI: 9-18) compared to young cattle (13-24 months of age) at 4% (95% CI: 1-6). Since newborns have a certain amount of maternal antibodies in their bodies, the interference of maternal antibodies cannot be ruled out by the method of antibody detection (Sun and Liu, 2019). Furthermore, calves may become infected from the mother *in utero* (Hasonova and Pavlik, 2006). Interestingly, several studies have shown that calves are more susceptible to MAP infection (Maruyama et al., 2003), which could be through the milk contaminated with MAP, or contact with feces containing MAP, in addition, it has been reported that adult cows and calves are equally susceptible to MAP, which is not quite what we expected (Nakada. et al., 2022). Unfortunately, only a small fraction of our statistical studies of MAP testing in calves have considered maternal antibody interference. Diagnostic methods and studies should be refined to enhance the accuracy of future PTB prevalence surveys.

In addition, the average herd size is also an important factor in the pooling positivity of bovine PTB, and we find that the positivity is relatively higher for average herd sizes between 500 and 1,000 cattle than for herd sizes that include less than 500 or more than 1,000 cattle. Furthermore, a previous study observed a minimal association between average herd size and infections by mycobacterial species other than MAP (Osterstock et al., 2008). However, Corbett et al (Corbett et al., 2018). reported that larger herds (>200 cows) were more likely to be MAP-positive than smaller herds. This observation is consistent with the results of our study, where the prevalence of MAP-positive is higher in medium-sized herds compared to smaller herds.

Dairy cattle had a higher prevalence of PTB (8%, 95%CI: 10-12) compared to dual-purpose cattle (3%, 95%CI: 1-5) and beef cattle (6%, 95%CI: 4-9), possibly due to the limited number of studies on PTB in Chinese dual-purpose cattle (yaks) (Mametimin., 2022). In addition, susceptibility to MAP infection can also be genetically influenced. It has been found that worldwide, the incidence of PTB is lower in beef cattle than in dairy cattle (Roussel, 2011), which is consistent with the findings of our study. This discrepancy might be attributed to the shorter feeding cycles of beef cattle compared to the longer cycles in dairy cows, potentially increasing infection risks (Cheng et al., 2016). At the same time, farming practices have an impact on PTB prevalence. In addition, most highland yaks are raised free-range, whereas dairy and beef cattle are usually intensively farmed at greater densities than dual-purpose cattle (yaks). This study analyzed the rates of bovine PTB infection in dairy, dual-purpose cattle, and beef cattle in China; however, due to the small number of studies on bovine PTB in dual-purpose cattle (yaks) in China, the results on yaks may have limitations, although the results nevertheless provide important reference data for the study of dual-purpose cattle PTB infection. For a certain period of time, our survey data on the true prevalence of cattle in each region is informative for the region’s government.

This meta-analysis offers a comprehensive review of PTB infection in Chinese cattle. Several limitations must be acknowledged. First, the 62 papers included were sourced from nine large databases, with not all applicable data points contributing to a lack of qualified literature. Also relevant studies in databases such as Scopus were not filtered into the manuscript, thus creating some limitations Second, the small sample sizes in the included studies may have contributed to unstable results in overall estimates and subgroup analyses. In general, differences in the results obtained by different authors may be influenced by factors such as the stage of infection, age of the animal, level of shedding of the organism, whether or not lactation is occurring, antibody concentration, and the sensitivity of different ELISA (Corbett et al., 2018). Therefore, factors such as organism shedding levels, antibody concentration, and maternal antibody interference should be considered when comparing PTB prevalence results. The lack of comprehensive literature limited this analysis, omitting some potential risk factors, including the failure to exclude maternal antibody interference in calves, which may introduce false positives. Despite these limitations, the meta-analysis sheds light on the overall prevalence and trends of PTB infections in China during the survey period.

## 5 Conclusion

In summary, this systematic review and meta-analysis revealed a pooled estimate of overall MAP positivity of 9% in the Chinese cattle herd during the 1981-2022 period. Moreover, significant PTB positivity variability is observed between regions and provinces. Age, average herd size, type of use, and season were important potential risk factors associated with PTB positivity. In addition, the detection method and sample type can also potentially affect the incidence of detected PTB. All in all, our study benefits veterinary practice, disease control, and policy implications, especially regarding control programs for paratuberculosis in Chinese bovine populations.

## Data Availability

The original contributions presented in the study are included in the article/supplementary material. Further inquiries can be directed to the corresponding author/s.
